# Osteogenic potential of apical papilla stem cells mediated by platelet-rich fibrin and low-level laser

**DOI:** 10.1007/s10266-023-00851-8

**Published:** 2023-10-24

**Authors:** David Gutiérrez Ramírez, Carolina Inostroza, Mahmoud Rouabhia, Camilo Alfonso Rodriguez, Lina Andrea Gómez, Mónica Losada, Ana Luisa Muñoz

**Affiliations:** 1 Buccal Innovation Research Group, Faculty of Dentistry, Universidad Antonio Nariño, Popayán, Colombia; 2 Facultad de Odontología, Universidad de los Andes, Santiago, Chile; 3 Faculté de Médecine Dentaire, Université Laval, Québec, Canada; 4 Faculty of Dentistry. Research Group of Oral Health, Universidad Antonio Nariño, Bogotá, Colombia; 5 School of Medicine, Biomedical Research Center (CIBUS), Universidad de La Sabana, Chía, Colombia; 6 Cellular and Functional Biology and Biomolecule Engineering Research Group, Faculty of Science, Universidad Antonio Nariño, Bogotá, Colombia; 7 Fundación Banco Nacional de Sangre Hemolife, Calle 23 No. 116-31, Bodega 26. Parque Industrial Puerto Central, Bogotá, Colombia

**Keywords:** Human Apical papilla stem cells, Low-level laser  therapy, Platelet-rich fibrin, Oral regeneration, Osteo-differentiation

## Abstract

To evaluate the osteogenic potential of platelet-rich fibrin (PRF) and low-level laser therapy (LLLT) on human stem cells from the apical papilla (SCAP) we isolated, characterized, and then cultured in an osteogenic medium cells with PRF and/or LLLT (660 nm, 6 J/m2-irradiation). Osteogenic differentiation was assessed by bone nodule formation and expression of bone morphogenetic proteins (BMP-2 and BMP-4), whereas the molecular mechanisms were achieved by qRT-PCR and RNA-seq analysis. Statistical analysis was performed by ANOVA and Tukey’s post hoc tests (*p* < 0.05* and *p* < 0.01**). Although PRF and LLLT increased bone nodule formation after 7 days and peaked at 21 days, the combination of PRF + LLLT led to the uppermost nodule formation. This was supported by increased levels of BMP-2 and -4 osteogenic proteins (*p* < 0.005). Furthermore, the PRF + LLLT relative expression of specific genes involved in osteogenesis, such as osteocalcin, was 2.4- (*p* = 0.03) and 28.3- (*p* = 0.001) fold higher compared to the PRF and LLLT groups, and osteopontin was 22.9- and 1.23-fold higher, respectively (*p* < 0.05), after 7 days of interaction. The transcriptomic profile revealed that the combination of PRF + LLLT induces MSX1, TGFB1, and SMAD1 expression, after 21 days of osteogenic differentiation conditions exposition. More studies are required to understand the complete cellular and molecular mechanisms of PRF plus LLLT on stem cells. Overall, we demonstrated for the first time that the combination of PRF and LLLT would be an excellent therapeutic tool that can be employed for dental, oral, and craniofacial repair and other tissue engineering applications.

**Supplementary Information:**

The online version contains supplementary material available at 10.1007/s10266-023-00851-8.

## Introduction

Dental stem cells are mesenchymal cells that include human dental pulp stem cells, gingival mesenchymal stem cells, stem cells from exfoliated deciduous teeth, periodontal ligament stem cells, dental follicle stem cells, stem cells from the apical papilla, and tooth germ stem cells. These stem cells are easy to obtain, with limited ethical challenges. These cells express mesenchymal, embryonic, neural, and vascular surface markers. They exhibit immunomodulation properties and multilineage differentiation, making them an important tool for regenerative medicine strategies for dental and other somatic tissues [1].

Because stem cells from the apical papilla (SCAPs) present a higher proliferation and mineralization potential compared with other dental stem cells [2], they are a promising tool for the regenerative medicine approach [3]. SCAPs are easy to extract from the apical papilla tissue of immature teeth, such as premolar or third molars obtained for pre-orthodontic treatment [4]. They exhibit differentiation capacities of osteogenic, adipogenic, odontogenic, neurogenic, and chondrogenic cell lineages [5, 6]. The SCAPs’ regenerative potential has been evaluated alone, with chemical and physical cell growth inductors, and in combination with biomaterials such as PRF as scaffolds [6, 7].

The platelet-rich fibrin (PRF) is a platelet concentrate with a three-dimensional structure that constitutes a fibrin matrix rich in several biomolecules, such as growth factors, cytokines, immunomodulators, among others, that are slowly released, stimulating cell proliferation and differentiation [8, 9]. The PRF was reported to promote stem cell proliferation and differentiation in a dose-dependent manner. Such effects were attributed to the release of different bioactive molecules, including transforming growth factor (TGF), platelet-derived growth factor (PDGF), and vascular endothelial growth factor (VEGF), as well as matrix proteins such as thrombospondin-1, fibronectin, and vitronectin [10–14].

For several decades now, low-level laser therapy (LLLT) has been used as a tool in clinical practice to manage different therapeutic processes [15]. LLLT was reported to increase the proliferation rate of different cell types, including stem cells [16, 17]. However, although LLLT has been widely used in vitro [18] and in clinical cases to stimulate tissue healing [19, 20], its complete mechanisms of action on target cells is not entirely understood. Some available studies suggest that LLLT therapy acts directly in the mitochondria by energy production in different cell types, including stem cells [21]. LLLT modulates the activity of specific transcription factors (the phosphorylation of tyrosine kinase receptors, nuclear factor kappa B), and increase the nitric oxide and growth factors (TGF-b, VEGF, etc.) production, thus influencing different cellular functions involved in cell proliferation [22]. Nevertheless, the effectiveness of LLLT depends on several parameters; thus, it is necessary to standardize the protocols of energy, intensity, and wavelength before its appropriate application [23, 24].

Since PRF and LLLT, separately, have been shown to promote stem cell proliferation and differentiation, we hypothesize that their combination could generate a synergic effect leading to better tissue regeneration. This study aimed to evaluate the impact of combined therapy of PRF plus LLLT on the osteogenic properties of human SCAPs.

## Materials and methods

### Isolation and characterization of SCAPs

The study was approved by the Ethics Committee of the Universidad Antonio Nariño (code: 06252018), and all the participants responded voluntarily to an informed consent form, according to the Helsinki Declaration. Blood and teeth were taken from healthy volunteers, no smokers or drug consumption, and blood analysis with normal ranges results. The teeth were collected from patients who required pre-orthodontic surgery for their dental treatments, and the radiography analysis was used to determine the Nola classification.

The apical papilla tissues were obtained from four third molars, extracted for pre-orthodontics treatment, without complete radicular development (Nolla Stage 8 and 9) of three volunteer participants, with a mean age of 17.5 ± 0.6 years. As previously reported, SCAPs were isolated and cultured by the explant culture method [17]. Cells were maintained in Dulbecco's modified Eagle medium (DMEM) (Lonza® High glucose), supplemented with penicillin/streptomycin (10,000 IU penicillin, 10,000 μg/ml streptomycin), l-glutamine (0.1 μM), and fetal bovine serum (10%) (Gibco®) until their ~ 85% confluence. In this study, we used cells between the 2 and 5 passages. For the SCAPs characterization, the cell adhesion and morphology were evaluated using optical microscope observations. A colony formation unit (UFC) assay was performed in 6-well tissue culture plates, and the colonies (≥ 50 cell aggregates) were scored.

Additionally, for chondrogenic and osteogenic differentiation, cells were incubated with chondrogenic [25], and osteogenic culture media [26]. Chondrogenic differentiation was evaluated by Alcian blue staining and the osteogenic differentiation with Alizarin red staining after 14 days. The immunophenotype was determined by immunostaining using specific monoclonal antibodies for CD90 (eBioscience # 12-0909-42), CD105 (Sigma-Aldrich # SAB4700259-100), CD34 (LifeSpan Bioscience #LS C20 4500), and CD 45 (eBioscience # 11-9459-42), cell acquisition was performed in a flow cytometry (BD Accuri ™ C6 flow cytometer). Samples were analyzed using FlowJo™ Software. Data from three independent experiments were acquired.

### PRF preparation

Peripheral blood of a healthy participant (25 years old) was collected in tubes without anticoagulant (Vacutainer® system, Becton Dickinson) that immediately were centrifuged (SCILogex® DMO 412) at 3200 rpm for 12 min, as previously we reported [27]. The PRF membrane [28] was sectioned in two parts (4 × 12 mm), and was placed into transwell inserts (pore of 0.4 μm Costar®) to avoid cell adhesion.

### Stem cell exposure to LLLT stimulation and PRF

Stem cells were seeded into 12-well plates and cultured for 24 h to allow cell adhesion. They were then exposed once to LLLT as previously described [17]. Briefly, we applied a Diode laser using a therapeutic laser lamp (DMC Therapy-XT®), emitting red radiation at a wavelength of 660 nm, spot size 15 mm with circular shape of the laser beam, energy density of 6 J/cm^2^, pulse frequency of 60Hz, in a continuous mode. The equipment was fixed to a base and placed perpendicularly in contact with the plate’s lid (angle of 90°) to warranty the irradiation conditions. In the PRF + LLLT group, we irradiated the cells and then put the transwell inserts with the PRF sections inside.

### Evaluation of osteogenic potential

The cultures were divided into four experimental groups. The first group refers to SCAPs stimulated with PRF. The second group refers to SCAPs stimulated with LLLT. The third group refers to SCAPs stimulated with PRF + LLLT. Finally, the fourth group refers to SCAPs without any stimuli (control group). In each group, the stem cells (1 × 10^4^ SCAPs/mL) were seeded into 12-well plates in DMEM supplemented with 10% fetal bovine serum and cultured for 24 h to allow cell adhesion. At the end of this culture period, the medium was changed in all experimental groups to osteogenic medium [26]. The osteogenic medium was replaced every 3 days. The osteogenic potential of SCAPs was assessed at different periods of time (7, 14, and 21 days).

### Evaluate bone nodule formation

The Alizarin red staining was performed at baseline, 7, 14, and 21 days after the exposure to PRF, LLLT, or PRF + LLLT. For this, the cultures were fixed with 70% ethanol, then overlaid with Alizarin red solution for 30 min. The cultures were then washed extensively, observed under an optical microscope, and photographed (Leica DM300). The nodules of mineralization in each microscope field (> 0.04 mm^2^) were counted.

Secretion of BMP-2, BMP-4 proteins

The supernatant medium of each experimental condition was collected at 24 h, 7, 14, and 21 days. According to the manufacturer's instructions, enzyme-linked immunosorbent assays (ELISA) were used to quantify BMP-2 and BMP-4 levels (Quantikine® ELISA R&D Systems—DBP200 and DBP400, respectively).

### Gene expression of osteoblastic markers

#### RNA-seq analysis of the differentiated SCAPs transcriptomic profile

Total RNA was extracted (RNeasy Mini kit, Qiagen) 21 days after cells were cultured in osteogenic medium in the presence or absence of PRF, LLLT, or PRF + LLLT. The RNA quality and yield were determined by NanoDrop spectrophotometer. Samples presenting RNA integrity number ≥ 7 and a 28S/18 ≥ 1.0 ratio were considered suitable for transcriptome sequencing. cDNA libraries were prepared with 1 μg of starting total RNA and sequenced using a DNBseq system with 100 bp paired-end reads length (Beijing Genomic Institute, BGI).

#### Quantitative RT-PCR (qRT-PCR) analyses

The gene expressions of relevant osteoblastic markers, including osteocalcin (OCN), osteopontin (OPN), and type 1 collagen (COL 1), were analyzed at 7, 14, and 21 days of incubation in an osteogenic medium in the presence or absence of PRF, LLLT, or PRF + LLLT by qRT-PCR. Briefly, total RNA from all experimental groups was extracted (RNeasy Mini kit, Qiagen) and reverse transcribed with MultiScribeTM RTranscripase (Thermofisher®), according to the manufacturer’s instruction. The amplicons were generated using primers listed in Table 1. Expression levels were estimated in duplicate, and phosphoglycerate kinase (PGK) was used as a normalization gene. ΔΔCt method was used to quantify the relative expression of each target gene.

**Table 1:** List of primers used for expression analysis by qRT-PCR

Gene	PRIMER SEQUENCE (5´-3´)	
	Forward	Reverse
OCN	CAT GAG AGC CCT CAC A	AGA GCG ACA CCC TAG AC
OPN	ACC AGA GTG CTG AAA CCC A	TGT GGA ATT CAC GGC TG
COL I	TGG AAA GAG CAG CCT CC	CGT TCT GTA CGC AGG TGA T
PGK	CGG GTC GTT ATG AGA GTC G	AAT TTG ATG CTT GGG ACA GC

### Statistical analysis

The normality of the data was determined with Shapiro–Wilk test. Data were presented as means ± standard deviations of at least three independent experiments. A one-way ANOVA test with Tukey and Dunnet post hoc test was performed for comparisons. Paired student *t* test was used for intragroup comparison. *p* values < 0.05 (*) and *p* < 0.01 (**) were considered significant. The statistical analyses were performed by the statistical package SPSS V21 (IBM, Armonk, NY, USA).

## Results

### Apical-papilla-derived cells exhibit stem cell phenotype

**Fig. 1:**
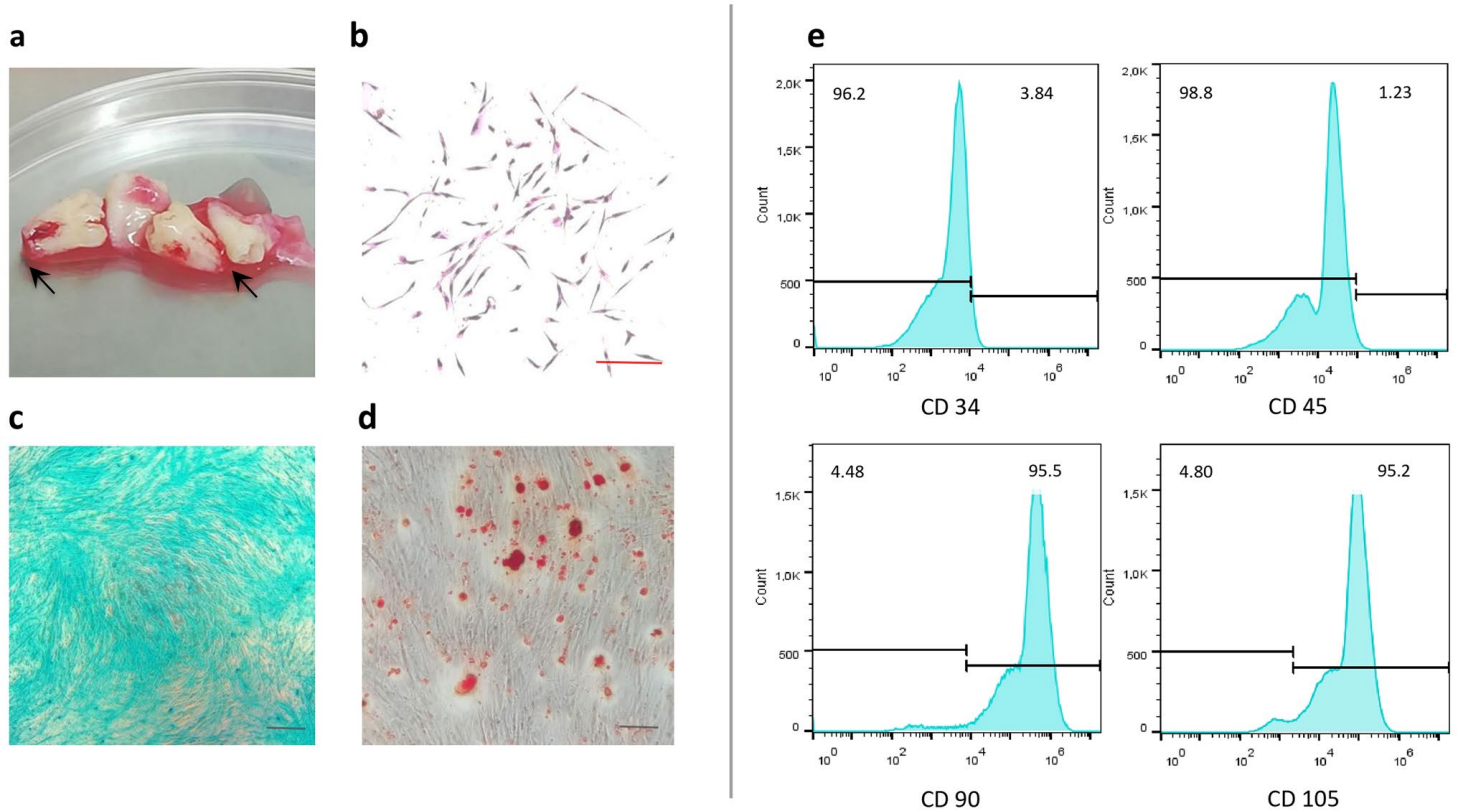
SCAP expansion and characterization. **a** The SCAP was obtained from third molars with uncompleted root formation. The apical papilla (black arrows) was extracted, and stem cells were obtained by an explant method. **b** Colonies formation capacity and cell morphology, bar indicates 10 µm; **c** chondrogenic differentiation is detected with Alcian blue staining, **d** osteogenic differentiation is shown with Alizarin red staining, bars indicate 50 µm; **e** representative flow cytometry histograms display the immunophenotype profile of SCAPs, including CD34^−^, CD45^−^, CD90^+^, and CD105^+^ in comparison with the control threshold (black lines)

The SCAPs were extracted from 9 of 12 teeth with apical papilla (Fig. 1a) and cultured by explant. The extracted cells showed colony formation unit, plastic adherence, auto-renewal capacity, and morphology as fibroblast-like cells (Fig. 1b). The differentiation assays showed that SCAPs could differentiate into chondrogenic and osteogenic lineages (Fig. 1c, d).

All populations (*n* = 3) were characterized by cluster of differentiation (CD) surface antigens and were homogenous. The immunophenotype revealed that SCAPs were positive for the mesenchymal stromal cell markers CD90 (95.5%) and CD105 (95.2%) and showed a minimal expression of hematopoietic stem cells markers CD34 (3.84%) and CD45 (1.23%) (Fig. 1e), confirming the stem cell phenotype of the extracted SCAPs.

### LLLT and PRF promote mineralized nodule formation by SCAPs

**Fig. 2:**
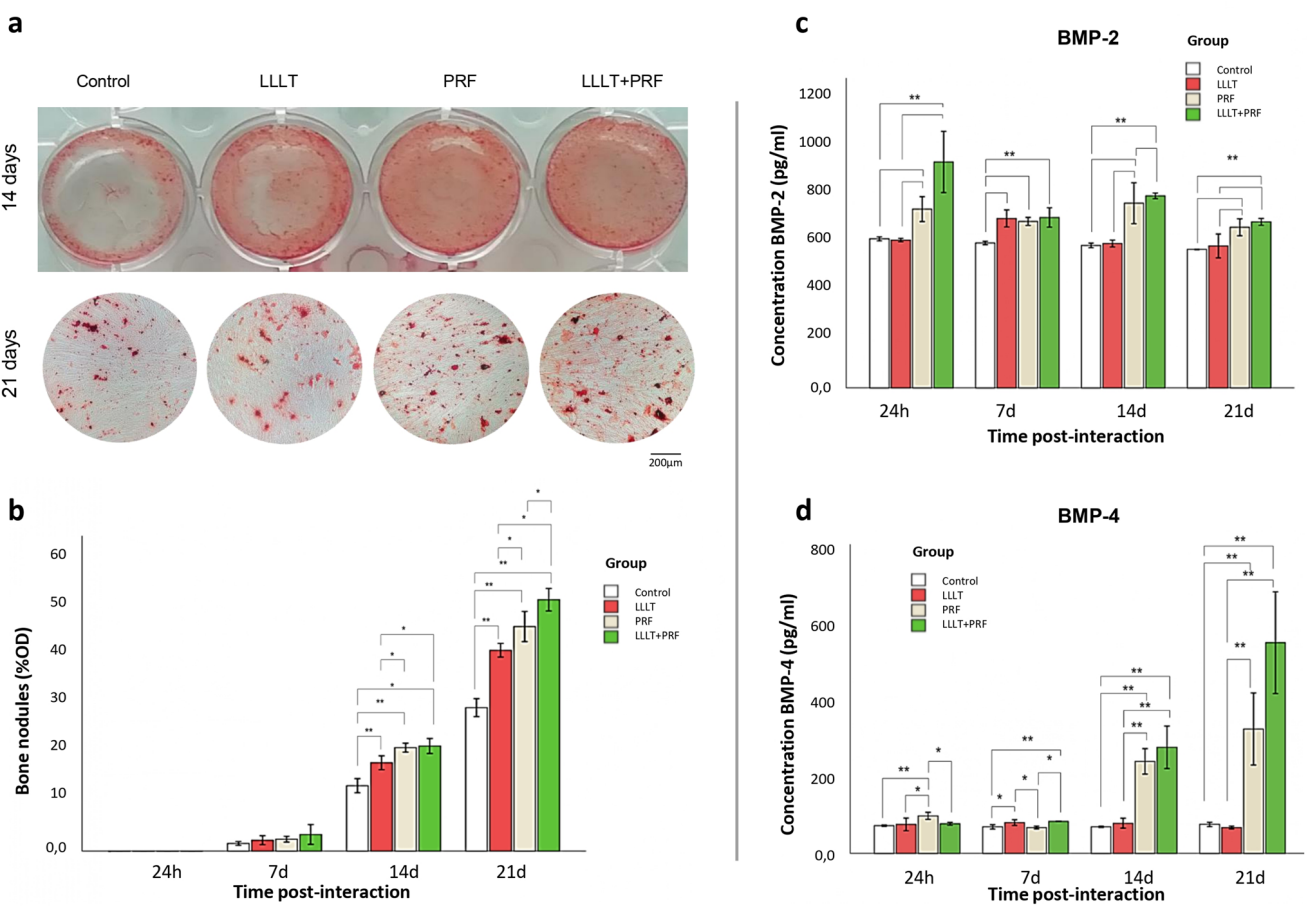
The osteogenic potential of stem cells from apical papilla under LLLT and PRF induction. **a** The Alizarin red staining at 14 and 21 days revealed an increase in osteogenic differentiation in experimental groups. **b** Bar graph represents the relative quantification of the mineralization by the optical density of nodules performed with Image J software. **c** Determination of BMP-2 and **d** BMP-4 at 1, 7, 14 and 21 days post-interactions shows higher levels in experimental groups than in the control group. (*) *p* < 0.005, (**) *p* < 0.001

Many mineralized nodules were observed with all culture conditions, including PRF, LLLT, and PRF + LLLT. However, the macroscopic evaluation showed differences in all groups (Fig. 2a). The quantitative analysis showed significant differences between all groups compared to the control at 14 and 21 days. Interestingly, cells cultured in the presence of LLLT + PRF showed the highest calcium nodule deposition at 21 days (*p* < 0.001) (Fig. 2b), suggesting the usefulness of such a combination in promoting bone regeneration.

### Increased secretion levels of BMP-2 and BMP-4 by SCAPs with PRF and LLLT

The bone BMP-2 expression was significantly higher in all groups compared to the control (*p* < 0.05). Furthermore, its expression was significantly higher in the PRF and LLLT + PRF groups (*p* < 0.05) at 24 h, 14, and 21 days (Fig. 2c). The expression of BMP-4 increased slightly in the PRF group at 24 h and in the LLLT and LLLT + PRF groups at 7 days. At 14 and 21 days, a significantly higher expression (*p* < 0.05) was observed in the PRF and LLLT + PRF groups compared to the other two groups evaluated (Fig. 2d).

### Increased gene expression profiles of osteoinduction in stimulated SCAPs

**Fig. 3:**
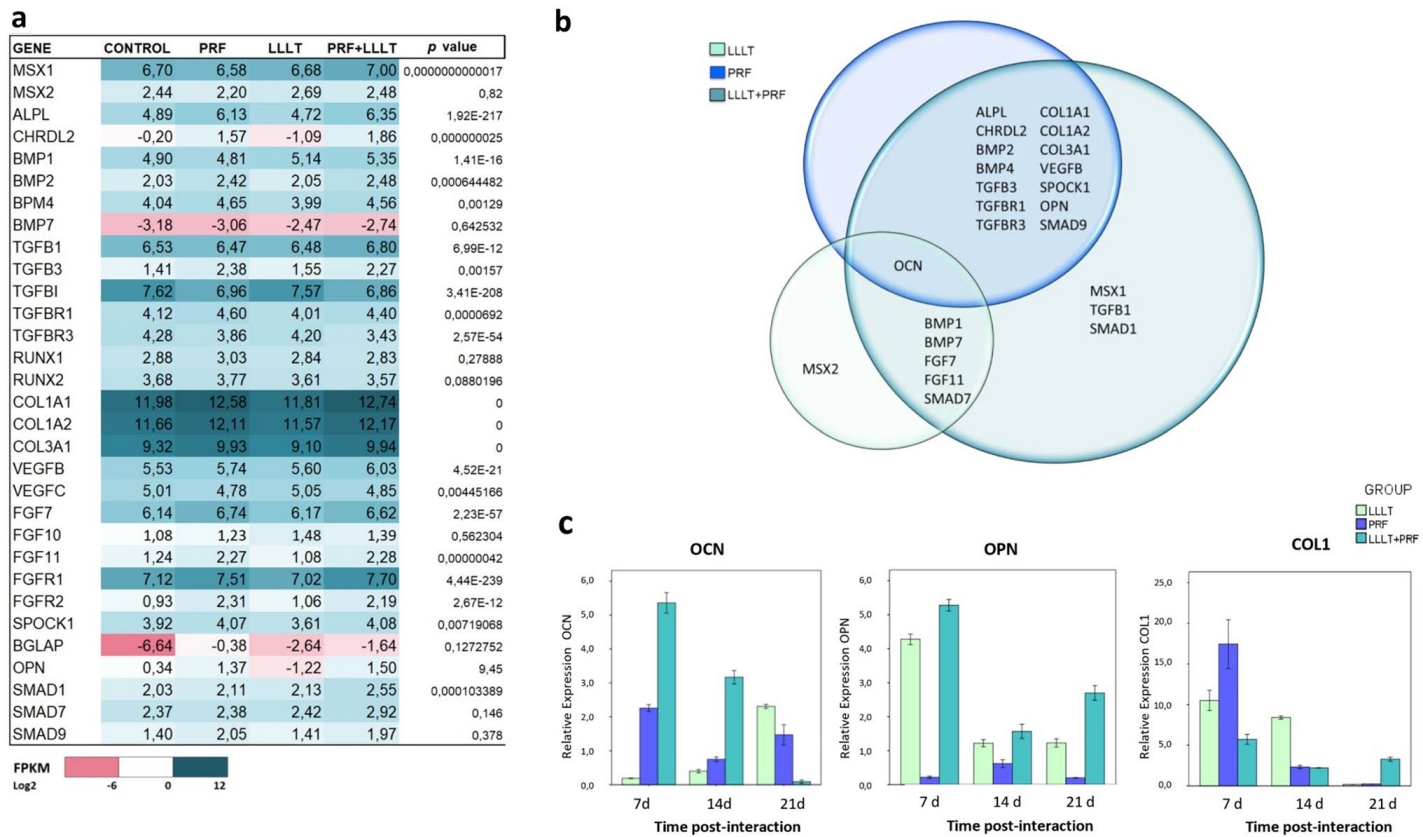
mRNA expression quantified by RNA-seq. **a** Heat map from osteogenic genes in stem cells from apical papilla (SCAP) stimulated with platelet-rich fibrin (PRF), low-level laser therapy (LLLT), PRF + LLLT, and control (osteogenic medium) at 21 days post-interaction. RNA-seq analysis was performed on samples collected from osteoinduced SCAP. Heat map was generated by Microsoft Excel® conditional formatting with a 3-scale color system [red (low) to blue (high)]. The color represents the FPKM (Log_2_). The *p* value corresponds to the comparison between control *vs.* PRF + LLLT. **b** Venn diagram shows the genes that exhibited the highest expression compared to the control. Relative mRNA expression levels by qRT-PCR. **c** The graphs show the expression of osteocalcin (OCN), osteopontin (OPN), and collagen type 1 (COL1) genes in SCAP stimulated with low-level laser therapy (LLLT), platelet-rich fibrin (PRF), and both (LLLT + PRF) at 7, 14, and 21 days post-interaction

The RNA-seq analysis showed an increase in the expression of osteogenic genes in all experimental groups compared to the control (Fig. 3a). Venn diagram displays subsets of genes that are regulated explicitly in each group. In the LLLT groups, BMP7, FGF7, FGF11, and SMAD7 genes were significantly overexpressed, but in the PRF groups, although more genes with increased expression being found than in the LLLT groups, ALPL, CHRDL2, BMP1, 2, and 4, and TGFBR1, among other genes, were significantly overexpressed, in comparison with the control group. Also, other genes showed the highest fragments per kilobase per million (FPKM) values only in the PRF + LLLT group, such as MSX1, TGFβ1, and SMAD1, compared with the other experimental groups. Of relevant interest is the intersection of the OCN gene that was upregulated during the osteoinduction in the three experimental groups (Fig. 3b).

### LLLT combined with PRF increased the expression of COL1 and OPN genes by SCAPs

The results demonstrated that in the SCAPs, PRF and/or LLLT stimuli upregulated the expression of OPN, OCN, and COL1 genes (Fig. 3c). Compared to the control, the mRNA expression of these three genes increased 7 days after interacting with PRF, LLLT, and PRF + LLLT. SCAPs cultured 7 days with PRF + LLLT exhibited a higher expression of OPN than those with only PRF and LLLT, which was 22.9- and 1.23-fold higher, respectively (*p* < 0.05). In the same way, PRF + LLLT also exhibited a higher OCN expression, which was 2.4-fold higher than PRF (*p* = 0.03) and 28.3-fold higher than LLLT (*p* = 0.001). Nevertheless, although COL1 expression was upregulated for the three groups, the highest expression level was shown by PRF, being 1.6-fold higher than LLLT (*p* = 0.002) and 2.9 -fold higher than PRF + LLLT (*p* = 0.001).

At 21 days, expression profiles of COL1 and OPN genes by qRT-PCR were consistent with the expression patterns revealed by the RNA-seq. PRF + LLLT induced a COL1 expression 19-fold higher than LLLT (*p* = 0.001) and 13.8-fold higher than PRF (*p* = 0.001), and an OPN expression 2.14fold higher than LLLT (*p* < 0.021) and 13.2-fold higher than PRF (*p* = 0.001) (Fig. 3a).

## Discussion

The regeneration or replacement of injured dental, oral, and craniofacial tissues is still an important challenge for treating damaged bone, cartilage, and tooth. The best tools to increase the efficiency of engineered constructs are still to be designed. Combining biomaterials, appropriate cells, cell growth factors and differentiation stimuli is an innovative topic in tissue regeneration. In this paper, we analyzed the effect of PRF and LLLT on osteo-differentiation of normal human SCAPs.

*PRF and LLLT promote osteogenic differentiation of SCAPs*

- Previously was evaluated the osteogenic potential of different concentrations of PRF exudate on stem cells derived from the periodontal ligament. They demonstrated that the PRF promotes the osteo-differentiation of these dental cells in dose-dependent manner, which was determined by the degree of mineralization and levels of ALPL activity and OCN gene expression [29]. In the same research line, Bi et al. (2020) demonstrated that the PRF improved the proliferation, differentiation, and migration of SCAPs. The osteo-differentiation was evaluated by Alizarin red test, which showed the highest nodule formation with PRF compared to SCAP not PRF exposed [30]. Similar to these previously reported results, our study showed a significantly higher relative percentage of mineralization nodules in the PRF groups than in LLLT and control groups

Additionally, the RNA-seq analysis showed that the stimulation of the SCAPs impacted osteogenic gene regulation. Interestingly the PRF group showed the largest number of overexpressed genes after 21 days of exposure, which included high FPKM values to osteogenic marker genes such BMP-2, OPN, COL1, SMAD9, ALPL. This could be explained by the presence of various growth factors in the PRF material, which promotes cell growth and differentiation [13, 14].

*SCAPs stimulated with PRF plus LLLT overproduce a possible synergic effect on osteogenic differentiation*

**Fig. 4:**
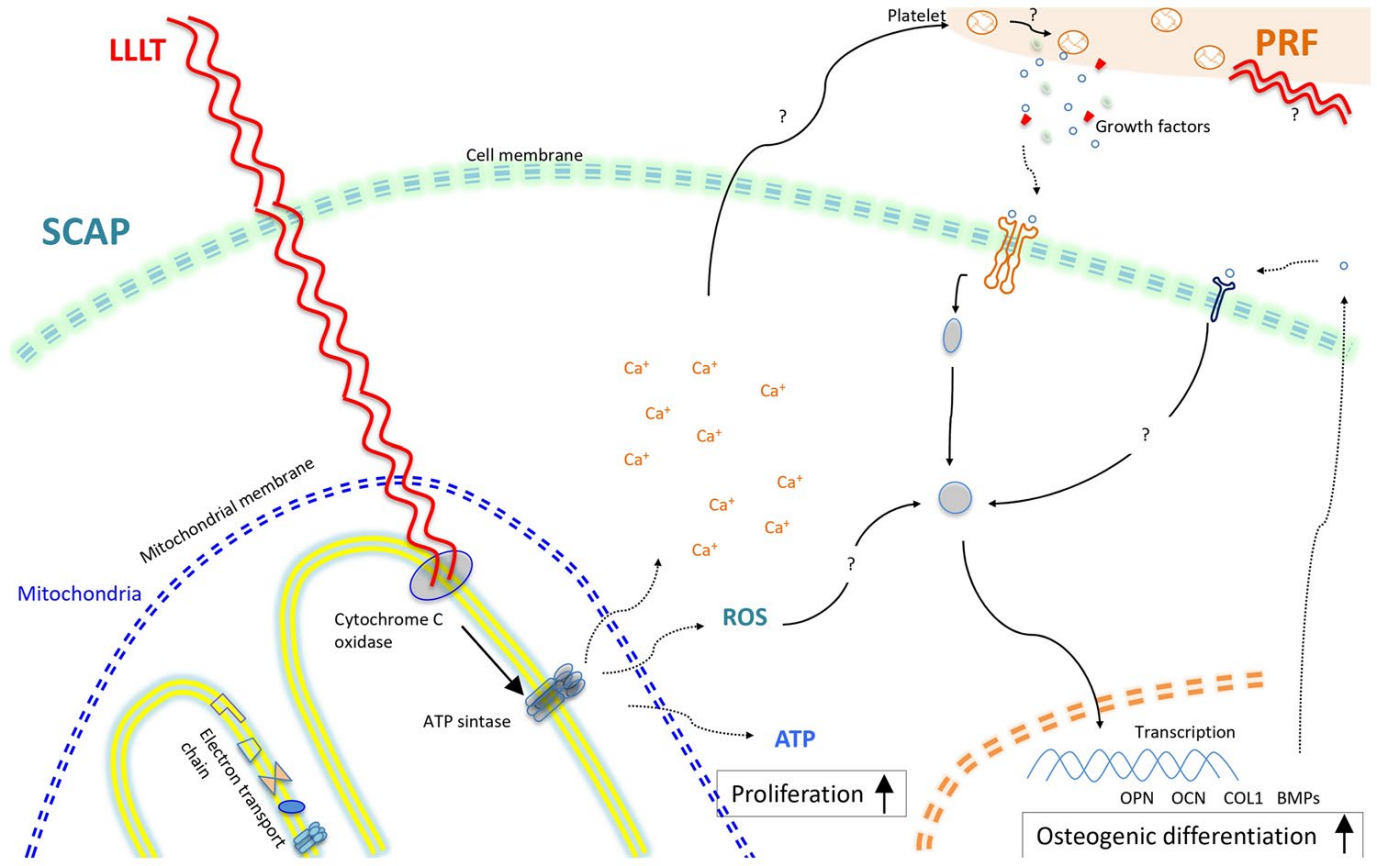
Schema of the hypothetical model of cellular and molecular interactions of the SCAP stimulated with platelet-rich fibrin and low-level laser therapy. LLLT and PRF may promote osteogenesis through different pathways, LLLT stimulating cell proliferation and PRF increasing the osteogenic differentiation potential

- We described that the stimulation of SCAPs with LLLT + PRF showed the highest values in most experimental tests, indicating two critical aspects. The first is that LLLT and PRF act synergistically over SCAPs, and the second is that each one may work in different pathways on the cells, or one may be empowering the other (Fig. 4).

However, there is still no available report about the potential mechanism of how PRF or LLLT regulates the osteogenic activity of SCAPs. About LLLT, this may be due to the photo-induction of cyclooxygenase enzime (COX), by the LLLT. Such activation could generate the necessary ions for the synthesis of ATP. This ATP increases energy availability, stimulates transcription, and promotes faster cell proliferation. Another possible way could be the effect of the LLLT on the intracellular concentration of the Ca + [22]; this ion could acts as a second messenger and interacts with intermediate proteins associated with pathways of osteo-differentiation (Fig. 4).

Similarly, we hypothesize that the PRF + LLLT interaction on SCAPs can generate stimulation in a double way. The LLLT-exposed cells can produce ions by cytochrome-c stimulation that, in turn, may activate the platelet degranulation, increasing the medium's growth factors. Further studies are needed to elucidate the role of these stimulation possibilities.

The RNA-seq analysis showed that the interaction of both stimuli (PRF + LLLT) promotes the activation of osteogenic genes such as MSX1, TGFΒ1, and SMAD1, which are not expressed in the groups that are stimulated with a single treatment or in control. These results are supportive of those previously reported, showing that the exposure of mesenchymal stem cells from different fonts to LLLT increased the expression of osteogenic genes, including RUNX2, ALP, OCN, OPN, BMP-2, BSP, COL1, CBFA, and others [31, 32]. The elucidation of this synergic mechanism will promote the clinical use of this treatment for tissue repair.

In clinical practice, both PRF and LLLT are used in different applications, but their application in combined use has not been proven. This study demonstrated that combining PRF and LLLT as an efficient therapy for dental, oral, and craniofacial repair is possible. Clinical studies with this approach are required as the advantages of each product (PRF or LLLT) may be potentiated, offering safe, and rapid treatments for the patient.

The main limitation of this study is the lack of in vivo experiments confirming the osteogenic activities of the PRF + LLLT, even so recently it has been reported that PRF + LLLT successfully promotes oral tissue regeneration in clinical conditions [33]; thus, we hypothesized that this combined therapy could be a tool for the management of bone defects promoting efficiently and safely the regeneration of lost or injured oral tissues.

In conclusion, this study demonstrated that the stimulation with platelet-rich fibrin or low-level laser therapy increased the osteogenic properties of SCAPS. Furthermore, this study showed for the first time that the combination of platelet-rich fibrin and low-level laser therapy promoted tissue regeneration better than PRF or LLT alone.

### Supplementary Information

Below is the link to the electronic supplementary material.

Supplementary Fig. 1 Methodological design scheme

## Data Availability

All data supporting the findings of this study are available within the paper.

## Declarations

### Cell lines

Human apical papilla stem cells were obtained and characterized as an aim of this study. Their use was approved by the ethical committee of the University Antonio Nariño (06252018).

## References

[1] Lei T, Zhang X, Du H (2021). Characteristics, classification, and application of stem cells derived from human teeth. Stem Cells Int.

[2] Bakopoulou A (2011). Comparative analysis of in vitro osteo/odontogenic differentiation potential of human dental pulp stem cells (DPSCs) and stem cells from the apical papilla (SCAP). Arch Oral Biol.

[3] Zhang W, Yelick PC (2021). Tooth repair and regeneration: potential of dental stem cells. Trends Mol Med.

[4] Sonoyama W (2008). Characterization of the apical papilla and its residing stem cells from human immature permanent teeth: a pilot study. J Endod.

[5] Smojver I (2022). Mesenchymal stem cells based treatment in dental medicine: a narrative review. Int J Mol Sei.

[6] Nada OA, El Backly RM (2018). Stem cells from the apical papilla (SCAP) as a tool for endogenous tissue regeneration. Front Bioeng Biotechnol.

[7] Wongwatanasanti N (2018). Effect of Bioceramic Materials on Proliferation and Odontoblast Differentiation of Human Stem Cells from the Apical Papilla. J Endod.

[8] Choukroun J (2006). Platelet-rich fibrin (PRF): a second-generation platelet concentrate. Part IV: clinical effects on tissue healing. Oral Surg Oral Med Oral Pathol Oral Radiol Endod.

[9] Dohan Ehrenfest DM (2010). Three-dimensional architecture and cell composition of a Choukroun's platelet-rich fibrin clot and membrane. J Periodontol.

[10] Dohan Ehrenfest DM (2009). In vitro effects of Choukroun's PRF (platelet-rich fibrin) on human gingival fibroblasts, dermal prekeratinocytes, preadipocytes, and maxillofacial osteoblasts in primary cultures. Oral Surg Oral Med Oral Pathol Oral Radiol Endod.

[11] Huang FM (2010). Platelet-rich fibrin increases proliferation and differentiation of human dental pulp cells. J Endod.

[12] Dohan Ehrenfest DM (2010). Choukroun's platelet-rich fibrin (PRF) stimulates in vitro proliferation and differentiation of human oral bone mesenchymal stem cell in a dose-dependent way. Arch Oral Biol.

[13] Wang J (2022). Effects of platelet-rich fibrin on osteogenic differentiation of Schneiderian membrane derived mesenchymal stem cells and bone formation in maxillary sinus. Cell Commun Signal.

[14] Strauss FJ (2020). Effect of platelet-rich fibrin on cell proliferation, migration, differentiation, inflammation, and osteoclastogenesis: a systematic review of in vitro studies. Clin Oral Investig.

[15] Hamblin MR (2016). Photobiomodulation or low-level laser therapy. J Biophotonics.

[16] Ginani F (2015). Effect of low-level laser therapy on mesenchymal stem cell proliferation: a systematic review. Lasers Med Sci.

[17] Gutierrez D (2021). Low-level laser irradiation promotes proliferation and differentiation on apical papilla stem cells. J Lasers Med Sci.

[18] Fernandes AP (2016). Effects of low-level laser therapy on stem cells from human exfoliated deciduous teeth. J Appl Oral Sci.

[19] Avci P (2013). Low-level laser (light) therapy (LLLT) in skin: stimulating, healing, restoring. Semin Cutan Med Surg.

[20] Lyu K (2022). The functions and mechanisms of low-level laser therapy in tendon repair (Review). Front Physiol.

[21] Hamblin MR (2018). Mechanisms and mitochondrial redox signaling in photobiomodulation. Photochem Photobiol.

[22] Rola PW, Lesiak S, Doroszko M, Włodarczak A (2022). Changes in cell biology under the influence of low-level laser therapy. Photonics.

[23] Eid FY (2022). A randomized controlled trial evaluating the effect of two low-level laser irradiation protocols on the rate of canine retraction. Sci Rep.

[24] Taylor DN, Winfield T, Wynd S (2020). Low-level laser light therapy dosage variables vs treatment efficacy of neuromusculoskeletal conditions: a scoping review. J Chiropr Med.

[25] Solchaga LA, Penick KJ, Welter JF (2011). Chondrogenic differentiation of bone marrow-derived mesenchymal stem cells: tips and tricks. Methods Mol Biol.

[26] Chen J (2013). Studies on culture and osteogenic induction of human mesenchymal stem cells under CO2-independent conditions. Astrobiology.

[27] Gutierrez DA et al. Evaluation of platelet rich fibrin obtained using different centrifugation parameters as a tool for regenerative medicine. Clin Lab. 2021;67(11). 10.7754/Clin.Lab.2021.210226 (**PMID: 34758218**) 10.7754/Clin.Lab.2021.210226 34758218

[28] Dohan Ehrenfest DM (2010). How to optimize the preparation of leukocyte- and platelet-rich fibrin (L-PRF, Choukroun's technique) clots and membranes: introducing the PRF Box. Oral Surg Oral Med Oral Pathol Oral Radiol Endod.

[29] Li X (2018). Platelet-rich fibrin exudate promotes the proliferation and osteogenic differentiation of human periodontal ligament cells in vitro. Mol Med Rep.

[30] Bi J (2020). Platelet-rich fibrin improves the osteo-/odontogenic differentiation of stem cells from apical papilla via the extracellular signal-regulated protein kinase signaling pathway. J Endod.

[31] Paschalidou M (2020). Biological effects of low-level laser irradiation (LLLI) on stem cells from human exfoliated deciduous teeth (SHED). Clin Oral Investig.

[32] Wang L (2019). Low-level laser irradiation modulates the proliferation and the osteogenic differentiation of bone marrow mesenchymal stem cells under healthy and inflammatory condition. Lasers Med Sci.

[33] Valamvanos K et al. The combined use of photobiomodulation therapy and platelet-rich fibrin for the management of two MRONJ stage II cases: an alternative approach. Frontiers Dent Med. 2022;3.

