# Supplementary figures and images for: Osteogenic potential of apical papilla stem cells mediated by platelet-rich fibrin and low-level laser
Source: Odontology. 2023 Oct 24;112(2):399–407. doi: 10.1007/s10266-023-00851-8 (PMC10925562; doi:10.1007/s10266-023-00851-8)

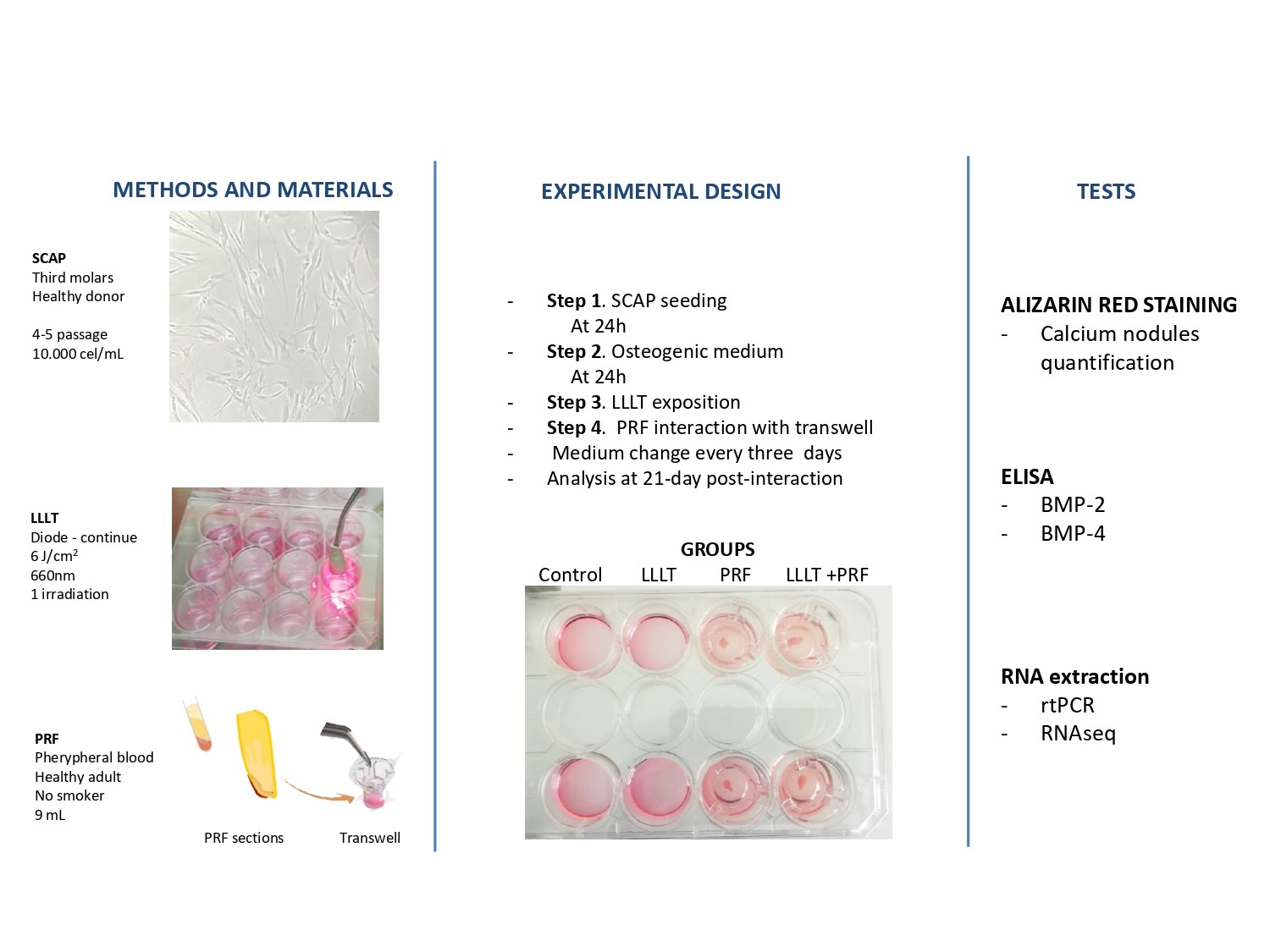

Supplement: Supplementary file 1 — Supplementary Fig. 1 Methodological design scheme [file 10266_2023_851_MOESM1_ESM.jpg]
